# The variability of the steps preceding obstacle avoidance (approach phase) is dependent on the height of the obstacle in people with Parkinson's disease

**DOI:** 10.1371/journal.pone.0184134

**Published:** 2017-09-14

**Authors:** Lucas Simieli, Lilian Teresa Bucken Gobbi, Diego Orcioli-Silva, Victor Spiandor Beretta, Paulo Cezar Rocha Santos, André Macari Baptista, Fabio Augusto Barbieri

**Affiliations:** 1 UNESP Univ Estadual Paulista, Faculdade de Ciências, Depto de Educação Física, Human Movement Research Laboratory, Bauru, São Paulo, Brazil; 2 UNESP Univ Estadual Paulista, Instituto de Biociências, Depto de Educação Física, Posture and Gait Studies Lab, Rio Claro, São Paulo, Brazil; University of Western Ontario, CANADA

## Abstract

Gait variability may serve as a sensitive and clinically relevant parameter to quantify adjustments in walking and the changes with aging and neurological disease. Variability of steps preceding obstacle avoidance (approach phase) are important for efficiency in the task, especially in people with Parkinson's disease (PD). However, variability of gait during the approach phase to obstacle avoidance in people with PD has been rarely reported, particularly when ambulating obstacles of different heights. The aim of the present study was to investigate the effects of obstacle height on step-to-step variability (step-to-step variability provides information on the variation between the "equivalent steps" for all trials, and walking variability (indicates the within-step variability of each, providing information about the modulations between the steps performed. of spatial-temporal parameters during the approach phase to obstacle avoidance in people with PD and neurologically healthy older people. Twenty-eight older people; 15 with PD and 13 neurologically healthy individuals (control group), participated in the study. Participants were instructed to walk at their preferred speed until the end of the pathway and to avoid the obstacle when it was present. Each subject performed 10 trials of the following tasks: unobstructed walking, low obstacle avoidance (3cm length, height equal ankle’s height, 60 cm wide), intermediate obstacle (3cm length, low plus high obstacle height divided by 2, 60 cm wide) avoidance and high obstacle avoidance (3cm length, knee’s height, 60 cm wide). The obstacle was positioned 4m from to the start position. The step-to-step and walking variability of the spatial-temporal parameters (acquiring with GAITRite^®^) of the four steps before obstacle avoidance were analyzed. MANOVAs were used to compare the data. PD group showed the characteristic gait deficits associated with PD. The obstacle increased the spatial-temporal variability (step–to-step and walking variability) during the approach phase to the obstacle. Specifically, both groups increased i) the step-to- step variability of the step length during low obstacle avoidance when compared to the other conditions; ii) the variability during low obstacle avoidance in the last step before obstacle (n-1) compared to higher obstacle avoidance; iii) variability during higher obstacle avoidance in further steps (n-3 and n-4). In conclusion, the presence of the obstacle during walking increased the variability of spatial-temporal parameters in older people with PD and the control group during the steps preceding obstacle avoidance. In addition motor planning (and motor adaptations) was initiated much earlier in the approach phase for the higher obstacle conditions compared to the low obstacle condition.

## Introduction

Previous studies have indicated that the steps preceding obstacle avoidance (approach phase) are important for efficiency in the task [1–3] especially in people with Parkinson's disease (PD) [4–5]. Any problems, such as poor balance or inadequate postural adjustments, during the approach phase may result in falls [4]. During the approach phase, actions are planned for safe obstacle avoidance according to the sensorimotor information obtained from (nearly) three steps preceding the obstacle [6]. In addition, people with PD present increased gait variability in complex tasks [7], such as obstacle avoidance, due to the cognitive deficits in this population during tasks that require divided attention [8]. Planning to avoid obstacles may be affected in people with PD due to sensorimotor deficits in this population [9], which can increase the variability of walking. Poor judgment about the way to avoid the obstacle is associate with this sensorimotor deficit, and exacerbates the variability [8,9,10]. Switching the center responsible for automatic gait control in people with PD makes walking less automatic and more dependent on cortical control, which seems to generate more variability in planning obstacle avoidance [10].

The variability of the temporal parameters of gait is related to mechanisms that regulate the movement rhythm and central pattern generator, while the variability of spatial parameters of gait is related to the balance control mechanisms [11]. Generally, gait variability has been analyzed in two ways in previous studies: i) walking variability and ii) step-to-step variability. Walking variability [7] indicates the within-step variability of each trial (i.e., variability of the steps n-4, n-3, n-2 and n-1 of the first trial), providing information about the modulations between the steps performed. This kind of variability represents all modulations that the subject performs during the task and is commonly used in research. Step-to-step variability [12]: provides information on the variation between the "equivalent steps" for all trials (i.e., step "n-1" of the first trial plus step "n-1" of the second trial, etc), which helps to analyze the online control (during performing the task [2]) of the individual, comparing, for example, the variability of step n-1 with the variability of step n-4 or another single step. This kind of variability could be helpful to understand the moment when modulations occur, and help the uptake of obstacle avoidance planning. According to Hausdorff and colleagues [12], a neurological deficit (e.g. Parkinson’s disease) increases variability and is related to falls. However, considering the demands and added complexity of walking in the real world when there are obstacles of varying type and size to negotiate as well as obstacles positioned at varying distances, variability of walking is fundamental to be able to adapt to varying environmental challenges. Thus, gait variability may serve as a sensitive and clinically relevant parameter to quantify adjustments in walking and changes with aging [13]. However, variability of gait during the approach phase to obstacle avoidance in people with PD has been rarely reported by previous studies.

The adjustments performed during the approach phase are dependent on the height of the obstacle in people with PD. Pieruccini-Faria [14–15] showed that people with PD present higher toe-clearance during high obstacle avoidance (20/25 cm of height) compared to low obstacle (5/10 cm), while for low obstacle avoidance, Vitório and collaborators [4] indicated that people with PD have a shorter stride duration during the approach phase to obstacle avoidance. However, both studies focus on the spatial-temporal parameters only and not variability, necessary variables to better understand the planning before obstacle avoidance. Therefore, the aim of the present study was to investigate the effects of obstacle height (low obstacle—5 or 10cm, -, intermediate obstacle—12.5, 15 or 17cm—and high obstacle avoidance—20 or 25cm) on step-to-step and walking variability of spatial-temporal parameters during the approach phase (steps n-4, n-3, n-2 and n-1 before the obstacle) to obstacle avoidance in older people with PD and neurologically healthy older individuals. The first hypothesis of the study was that older people with PD would show greater step-to-step and walking variability independent of the height of the obstacle when compared to neurologically healthy older individuals due to loss of automaticity of gait and increased voluntary command of gait [10]. Moreover, due to poorer perception and cognition in PD [9], the planning during the approach phase will be compromised. The second hypothesis of the study was that higher obstacle avoidance (20/25 cm) would increase the variability of spatial-temporal parameters during the approach phase to obstacle avoidance compared to other obstacle heights (low and intermediate obstacles). The higher obstacle will be more dangerous than the other and increase the number of adjustments before avoidance, increasing the variability [10]. Finally, the third, and last, hypothesis was that the steps closer to the obstacle (penultimate and final steps—n-2 and n-1, respectively) would show greater variability of spatial-temporal parameters compared to the steps farthest from the obstacle (steps n-3 and n-4), especially for older people with PD, regardless of the obstacle height. This hypothesis suggests that to the closer steps are more important for the last adjustments, and that decreased perception in older people with PD leads them to perform more adjustments closer to the obstacle to avoid the obstacle with greater confidence [1,2].

## Materials and method

The study was approved by the research ethics committee of the of the São Paulo State University at Rio Claro—Brazil (#580.665/2013). Individuals gave informed consent by signing the free and informed consent form approved by the local Ethics Committee.

The study included 28 individuals; 15 older people with PD (PD group—8 men and 7 women) and 13 neurologically healthy older individuals (control group—7 men and 6 women). An experienced neurologist evaluated and diagnosed the older people with PD according to the London Brain Bank guideline for diagnosis [16].

The following exclusion criteria were established for both groups: age under 60, cognitive decline, history of orthopedic problems, use of any walking aid, vision deficits (glaucoma, cataract) and vestibular problems (dizziness, labyrinthitis) that prevented performance of the experimental protocol. Participants with diabetes mellitus were also excluded, since plantar sensitivity may be altered and thereby, impair gait [17]. No individuals wore bifocal glasses, and all individuals that wore glasses that corrected their visual deficits, such as myopia, farsightedness, astigmatism, were included in the sample. In addition, for older people with PD, the individual was required to be under dopaminergic medication treatment and in a stage of PD up to III according to the Hoehn & Yahr disability scale [18–19]. Older people with PD were selected from the database of the Physical Activity Program for Patients with Parkinson's disease (PROPARKI—UNESP—Rio Claro). For the control group, neurologically healthy individuals were selected from the database of the Physical Activity Program for Older People (PROFIT—UNESP—Rio Claro).

The clinical and gait evaluations of the PD group were performed in an "ON" state of medication (about an hour after taking the dopaminergic medication). Individuals gave informed consent by signing the informed consent form approved by the local Ethics Committee (#580.665/2013).

### Clinical evaluation

To determine the degree and stage of disease, patients were evaluated using the Unified Parkinson's Disease Rating Scale—UPDRS [20] and the Hoehn and Yahr score [21], respectively. In addition, both groups had their cognitive function assessed by the Mini Mental State Examination (MMSE—(Score according to years of schooling, [22]).

### Experimental design

Each subject performed 10 trials of the following tasks: unobstructed walking, low obstacle avoidance, intermediate obstacle avoidance and high obstacle avoidance. Thus, each participant performed 40 trials in total. The trials were performed in blocks. The order of blocks started, for all participants, with the unobstructed walking, and, then, the other conditions were randomized for each participant.

Participants were instructed to walk at their preferred speed until the end of the walkway (8 m). In the trials where the obstacle was present, the obstacle was placed in the center of the walkway, 4 m from the start position, which was adjusted to ensure comfortable crossing with the right leg. The participants were instructed to avoid contact with the obstacle in trials where the obstacle was present. Obstacles were adjusted according to the subject’s ankle and knee height. The height of the low obstacle was 5 or 10cm when participant’s height ankle was lower or higher than 7cm, respectively. The height of high obstacle was 20 or 25cm when participant’s height knee was lower or higher than 48.5cm, respectively. The height of the intermediate was the ratio of low obstacle and high obstacle sum. The other dimensions of the obstacle were 60cm long and 3cm wide.

**Fig 1:**
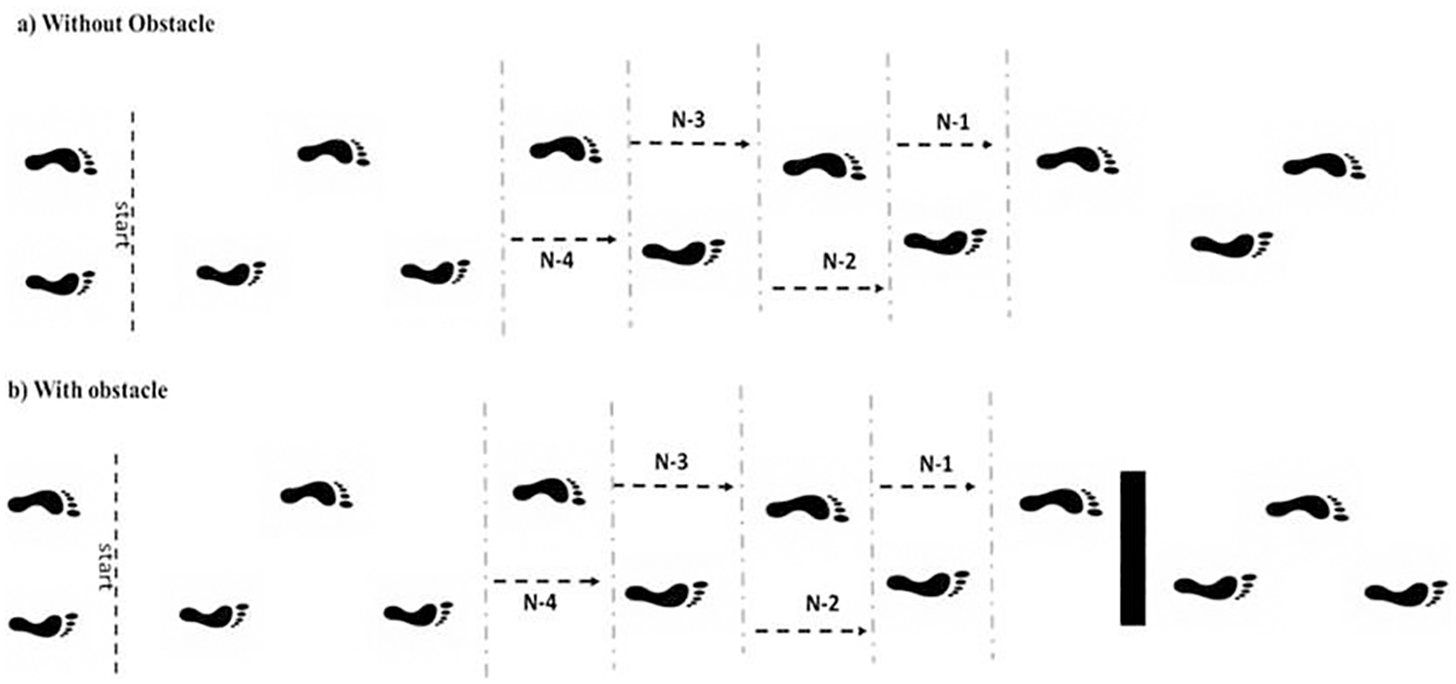
Representative illustration of the steps analyzed in unobstructed walking (a) and conditions with obstacle avoidance (b).

The spatial-temporal parameters were collected with a computerized mat (GAITRite^®^, CIR System, Clifton, NJ, USA) and three-dimensional optoelectronic system (OPTOTRAK Certus)–frequency 100 Hz. For unobstructed walking (Fig 1a), step length, width, duration and velocity and single and double support duration (calculated as a percentage of step duration) were analyzed for the four mid-pathway steps (after three or four steps from the start point). For the conditions with obstacle avoidance, the four steps preceding obstacle avoidance were analyzed (n-4, n-3, n-2 and n-1) (Fig 1b). The toe-clearance (mean values) were calculated for each limb (leading limb and trailing limb).

### Data analysis

The walking variability [7] and step-to-step variability [12] of the spatial-temporal parameters were analyzed. For walking variability, the average value of 4 step parameters for each trial was initially calculated. Next, the average and standard deviation for each participant in each condition were calculated. Finally, the coefficient of variation [13,23,24] of each participant and condition were calculated. For step-to-step variability, the average values and standard deviations for each step for each participant were calculated, followed by the coefficient of variation [13,23,24] of each step in each condition. In addition, for conditions with obstacle avoidance, the horizontal distance between the foot and the obstacle for each step was calculated [2] and the variability of this distance was analyzed. To calculate the variability of this distance, we used the coefficient of variation for each step.

### Statistical analysis

The data of interest was statistically analyzed using SPSS 15.0 software for Windows^®^ with a significance level maintained at 0.05. The Shapiro-Wilk and Levene’s tests were used to verify the normal distribution of data and homogeneity of variance, respectively. Participant characteristics and cognitive function were compared between groups using the Student's t-test for independent samples. The mean values and the walking variability of spatial-temporal parameters were compared through MANOVAs for group (PD group x control group) and condition (unobstructed walking x low obstacle x intermediate obstacle x high obstacle), with repeated measures for condition. The step-to-step variability of the spatial-temporal parameters was compared using MANOVAs with group and step (n-4 x n -3 x n-2 x n-1), with repeated measures for step. For variability of the horizontal distance from the foot to the obstacle, the parameters were compared using MANOVAs with factor for group and condition (low obstacle x intermediate obstacle x high obstacle), with repeated measures for condition. Tukey univariate tests were performed to identify differences when the MANOVA revealed significant interactions. Moreover, we’ve confirmed if they have present learning effect during the trials comparing one trial with the next, and they didn’t have significant difference.

## Results

The t-test demonstrated no significant differences between the groups for age, height, body mass or cognitive function (Table 1). The PD group presented a disease stage of 2.10 ± 0.40 pts (H&Y) and UPDRS-motor of 23.50 ± 9.41 pts (mild to moderate stage). There was no contact with the obstacle during obstacle avoidance trials.

**Table 1:** Means and standard deviations of anthropometric characteristics and cognitive function for the PD group and control group. MMSE: Mini Mental State Examination.

Group	Age (years)	Height (m)	Body weight (kg)	Mini Mental State Exam (pts)
**PD group**	70.66±6,55	1.63±0.07	70.61±9.82	28.26±1.66
**control group**	71.53±5.42	1.59±0.08	70.50±15.49	28.46±1.63
**p-value**	0.76	0.20	0.98	0.76

### Mean values of the spatial-temporal parameters

The MANOVA revealed a significant effect for group (Wilks' Lambda = 0.70, F_6,271_ = 18.58; p<0.001), condition (Wilks' Lambda = 0.06, F_18,259_ = 218.61; p<0.001) and interaction between factors (Wilks' Lambda = 0.74, F_18,259_ = 4.91; p<0.001).

For group (Table 2), the control group demonstrated greater step length and velocity and shorter double support duration compared to the PD group. For condition (Table 2), unobstructed walking showed greater length and step velocity and double support duration, narrower step width and shorter single support duration in comparison to all conditions with obstacle avoidance. In addition, the condition with low obstacle (both groups) avoidance presented slower step velocity compared to the other conditions with obstacle avoidance. Moreover, participants of both groups presented longer single support and double support duration when they performed high and intermediate obstacle avoidance compared to when they performed low obstacle avoidance.

**Table 2:** Means and standard deviations of spatial-temporal parameters for each condition.

	Control group				PD group			
	Unobstructed walking	Low obstacle	Intermediate obstacle	High obstacle	Unobstructed walking	Low obstacle	Intermediate obstacle	High obstacle
**Step length(cm)**	63.16±0.71	61.82±0.73	61.76±0.77	61.49±0.77	58.60±0.66	57.12±0.68	56.80±0.72	57.04±0.72
**Step duration (s)**	0.50±0.01*	0.54±0.04	0.51±0.05*	0.51±0.05*	0.54±0.00	0.55±0.05	0.54±0.02	0.54±0.01
**Step width (cm)**	10.63±0.25	11.57±0.29	11.09±0.28	11.39±0.30	10.86±0.23	11.44±0.27	11.63±0.28	11.83±0.30
**Step velocity (cm/s)**	125.60±1.69	118.19±1.68*	121.92±1.68	121.63±1.62	109.57±1.58	104.96±1.56	105.95±1.57	105.96±1.50
**Single support duration (%)**	37.79±0.16*^,^^#^	**41.47±0.24**^d^^,^^e^	**43.70±0.19**^f^	44.36±0.26	**36.74±0.15**^#^	**41.87±0.22**^d^^,^^e^	**43.46±0.18**^f^	44.63±0.24
**Double support duration (%)**	23.67±0.27*^,^^#^	21.79±0.30*^,^^d^^,^^e^	22.37±0.29*^,^^f^	22.82±0.29*	25.15±0.25	24.85±0.28	24.82±0.27	24.81±0.27

*—group difference,

^#^—difference between unobstructed walking and all conditions with obstacle avoidance,

^d^—difference between low and intermediate obstacle conditions;

^e^—difference between low and high obstacle conditions,

^f^–difference between

### Walking variability

The MANOVA revealed a significant effect for group (Wilks' Lambda = 0.27, F_6,83_ = 489.55; p<0.001), condition (Wilks' Lambda = 0.08, F_18,71_ = 127.09; p<0.001) and interaction between factors (Wilks' Lambda = 0.72, F_18,71_ = 50.58; p<0.001) for walking variability.

For group, the PD group demonstrated greater variability in step width (p<0.001), step velocity (p<0.003) and single support duration (p<0.001) than the control group. For condition, there was greater variability in step length when the obstacle was present, regardless of its height. In addition, for step duration, the low obstacle condition showed greater variability than the unobstructed walking (p<0.001) and high obstacle (p<0.003) conditions. For step width, unobstructed walking and low obstacle conditions presented greater variability than other obstacle height (intermediate—p<0.001 and high—p<0.001) conditions. Finally, variability of step velocity presented greater values for the intermediate obstacle condition in comparison to the unobstructed walking (p<0.001) and high obstacle (p<0.001) conditions.

**Fig 2:**
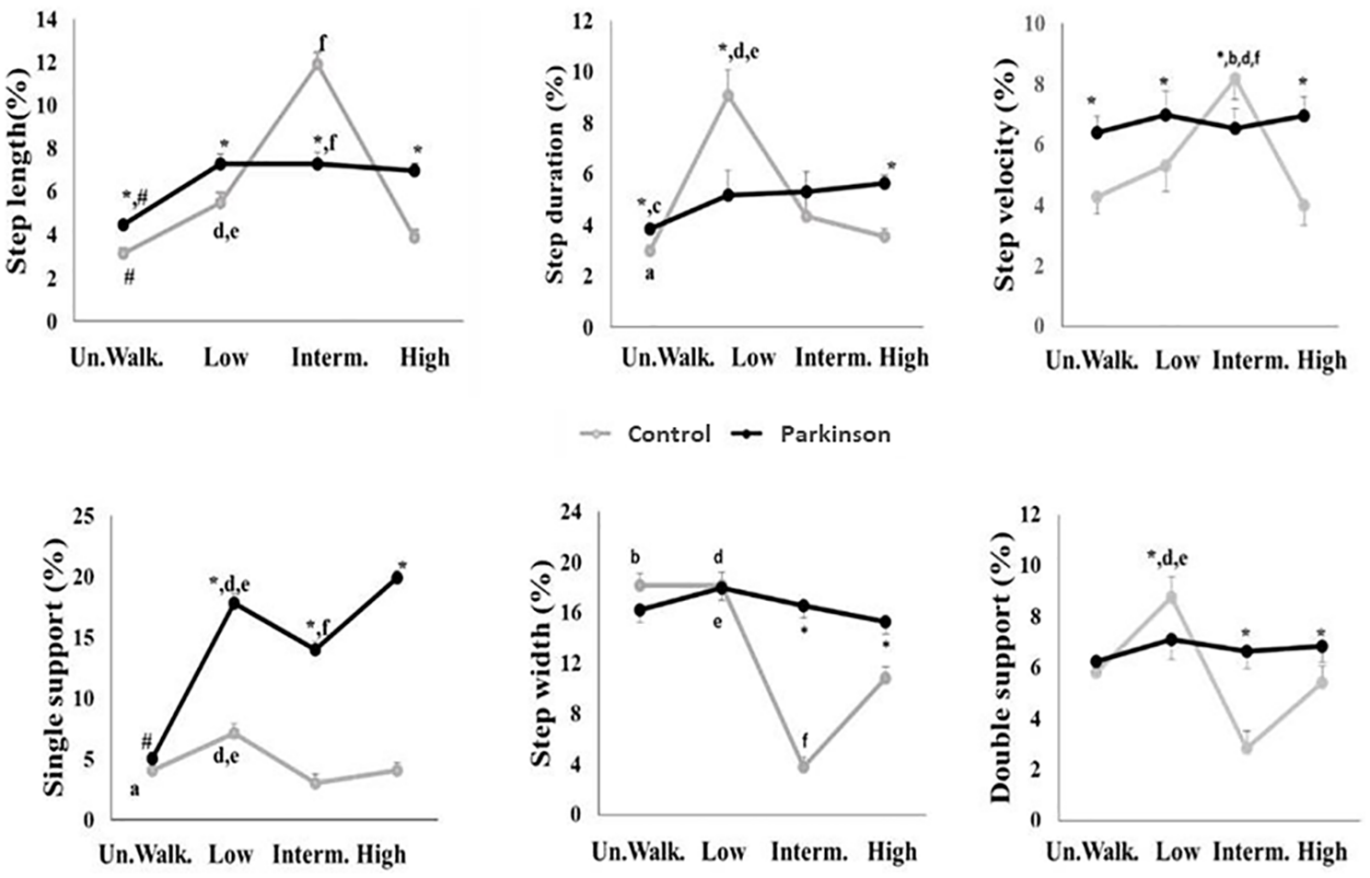
Means and standard deviations for walking variability of spatial-temporal parameters for each condition. Un.Walk.: unobstructed walking; Low: walking with low obstacle avoidance; Interm.: walking with intermediate obstacle avoidance; High: walking with high obstacle avoidance. p<0.05, *: Difference between groups, ^#^: difference between unobstructed walking and walking with obstacle avoidance, regardless of obstacle height; ^a^: difference between Un.Walk and Low; ^d^: difference between Low and Interm.; ^e^: difference between Low and High; ^f^: difference between Interm. and High.

For interaction between group and condition (Fig 2), the control group showed greater variability than the PD group for step length (p<0.001) and velocity (p<0.001) during intermediate obstacle avoidance. For the conditions with obstacle avoidance (low, intermediate and high obstacles), the PD group demonstrated higher variability for step width and single and double support duration compared to the unobstructed walking condition. The PD group presented higher values (p<0.05) of variability in all parameters compared to the control group (Fig 2), except for step length (p = 0.33) and duration (p = 0.99). Moreover, the control group showed greater variability of single and double support when they performed low obstacle avoidance in comparison with the other conditions.

### Step-to-step variability

For step-to-step variability, the MANOVA demonstrated a significant effect for group (Wilks' Lambda = 0.17, F_6,83_ = 65.55; p<0.001), step (Wilks' Lambda = 0.40, F_18,255_ = 4.98; p<0.001) and condition (Wilks' Lambda = 0.85, F_18,71_ = 42.38; p<0.001). In addition, there was an interaction between the group * step (Wilks' Lambda = 0.43, F_18,255_ = 4.46; p<0.001), group * condition (Wilks' Lambda = 0.72, F_18,71_ = 42.38; p<0.001), condition * step (Wilks' Lambda = 0.10, F_54,219_ = 4.41; p<0.001) and group * step * conditions (Wilks' Lambda = 0.09, F_54,219_ = 4.66; p <0.001).

Effects of group and condition are presented above. For the step factor (Table 3), the variability of step length and double support duration of the n-1 step were greater (p<0.001) compared to all other steps (n- 4, n- 3 and n-2 steps). In addition, n-1 steps showed greater variability of single support duration compared to the n-4 and n-3 steps (p <0.01). Furthermore, the n-2 step showed greater variability than the n-4 step for single support duration (p<0.001).

**Table 3:** Means and standard deviations of the variability of spatial-temporal parameters interaction group * step * condition.*

		Control Group				PD Group			
		Unobstructed walking	Low obstacle	Intermediate obstacle*	High obstacle	Unobstructed walking	Low obstacle	Intermediate obstacle*	High obstacle
**Step length (%)**	**n-4**	3.27±0.57	3.88±1.19	**13.62±1.34**^b^^,^^d^^,^^f^	3.83±0.84	4.92±0.57	7.74±1.19	9.02±1.34	8.18±0.84
	**n-3**	2.82±0.44	4.78±0.91	**11.98±1.02**^b^^,^^d^^,^^f^	3.26±0.64	4.37±0.39	7.18±0.81	8.01±0.91	7.17±0.57
	**n-2**	3.30±0.42	4.50±0.87	**11.69±0.98**^b^^,^^d^^,^^f^	3.38±0.61	4.29±0.39	7.15±0.81	5.24±0.91	7.05±0.57
	**n-1**	3.27±0.42	**8.81±0.87**^§^	**10.31±0.98**^b^^,^^d^^,^^f^	5.11±0.61	4.35±0.40	7.06±0.84	6.90±0.94	5.55±0.59
**Step duration (%)**	**n-4**	2.68±0.49	4.05±2.50	4.46±1.93	3.52±0.77	4.44±0.49	5.91±2.50	6.18±1.93*	6.73±0.77*
	**n-3**	3.41±0.38	7.55±1.90	2.30±1.47	3.17±0.59	3.47±0.34	4.97±1.87	5.62±1.30*	5.29±0.53*
	**n-2**	2.75±3.65	3.13±1.83	3.91±1.41	3.24±0.77	3.79±0.30	4.93±1.75	4.58±1.32	5.22±0.63*
	**n-1**	3.17±0.36	21.54±1.83§	6.74±1.41	4.21±0.57	3.66±0.35	4.89±1.76	4.85±1.36*	5.36±0.54*
**Step width (%)**	**n-4**	16.42±2.37	15.82±2.61	7.45±1.97	10.34±2.23	15.28±2.37	16.12±2.61	16.87±1.97*	13.31±2.23*
	**n-3**	16.98±1.81	18.17±1.99	3.16±1.51	12.74±1.70	16.25±1.62	18.67±1.78	17.52±1.35*	16.82±1.56*
	**n-2**	18.15±1.74	16.36±1.71	2.40±1.45	9.70±1.64	17.08±1.62	18.37±1.85	15.67±1.33*	17.26±1.52*
	**n-1**	21.12±1.74*	**22.26±1.91**^§^^,^*	1.94±1.49	10.39±1.64	16.38±1.68	18.73±1.84	16.20±1.39*	13.82±1.58*
**Step velocity (%)**	**n-4**	3.99±0.82	4.07±1.18	7.42±1.26	4.54±0.89	7.29±0.82	7.16±1.18	9.10±1.26	7.96±0.89
	**n-3**	4.67±0.63	5.33±0.90	8.57±0.96	3.51±0.68	6.06±0.56	7.01±0.89	7.76±0.86	7.16±0.67
	**n-2**	4.26±0.60	3.64±1.86	9.90±1.93	3.68±0.75	6.08±0.46	6.65±0.80	4.31±0.96	7.12±0.61
	**n-1**	4.20±0.60	8.13±0.86	6.86±1.97	4.23±0.62	6.15±0.58	7.04±0.83	4.99±0.89	5.59±0.63
**Single support (%)**	**n-4**	3.70±1.40	5.21±2.06	2.17±1.68	4.57±1.61	5.48±1.40	19.70±2.06*	24.67±1.68*	29.21±1.61*
	**n-3**	3.14±1.07	6.16±1.57	3.12±1.28	3.01±1.22	4.70±0.96	17.06±1.40*	21.22±1.14*	23.71±1.10*
	**n-2**	3.79±1.03	4.98±1.51	3.29±1.23	3.97±1.18	5.00±0.96	17.14±1.45*	5.43±1.24	22.39±1.20*
	**n-1**	5.71±1.09	**12.06±1.58**^§^	3.62±1.27	4.71±1.19	4.97±0.99	17.45±1.45*^.^^§^	4.79±1.18	4.24±1.13
**Double support (%)**	**n-4**	5.02±1.37	4.41±1.79	2.31±0.64	4.60±0.65	6.35±1.37	7.34±1.79	7.94±0.64	7.45±0.65
	**n-3**	5.18±1.05	8.30±1.37	2.47±0.48	5.05±0.50	6.13±0.94	7.01±1.22	6.88±0.43	7.35±0.44
	**n-2**	5.06±1.01	5.15±1.31	2.43±0.47	5.11±0.48	6.15±0.94	6.94±1.22	5.39±0.43	7.40±0.44
	**n-1**	7.90±1.01	**17.08±1.31**^§^	4.11±0.47	6.83±0.48	6.25±0.97	7.13±1.26	6.24±0.45	5.11±0.46

*:Difference between groups,

^b^–difference between unobstructed walking and intermediate obstacle conditions,

^d^–difference between low and intermediate obstacle conditions,

^f^—difference between intermediate and high obstacle conditions,

^§^—difference between n-1 and all other analyzed steps.

For interaction group * step * condition (Table 3), the PD group showed greater variability of step length and velocity and single and double support duration in the n-4, n-3 and n-2 steps for the intermediate and high obstacle conditions compared to the control group. In the low obstacle condition, the control group showed greater variability of step width in the n-1 step and lesser variability of single support duration in the n-2 step than the PD group. For group * step (Table 3), the PD group showed higher variability of single support duration in the n-4, n-3 and n-2 steps in comparison with the control group (p<0.001). Furthermore, only for the PD group, the variability of single support duration in the n-2 and n-1 steps was greater than the variability in the n-4 and n-3 steps. For step * condition (Table 3), the variability of step length in the n-1 step (p<0.002) was higher than the other steps (n-2, n-3 and n-4 steps) during low obstacle avoidance. On the other hand, when performing intermediate and high obstacle avoidance, the participants showed greater variability of single support duration in the n-4 (p<0.001) and n-3 (p<0.001) steps when compared to the n-1 step. Moreover, the variability of step length, duration and width in the n-1 step during low obstacle avoidance was greater than during intermediate and high obstacle avoidance.

### Variability of the horizontal distance between the foot and the obstacle

**Fig 3:**
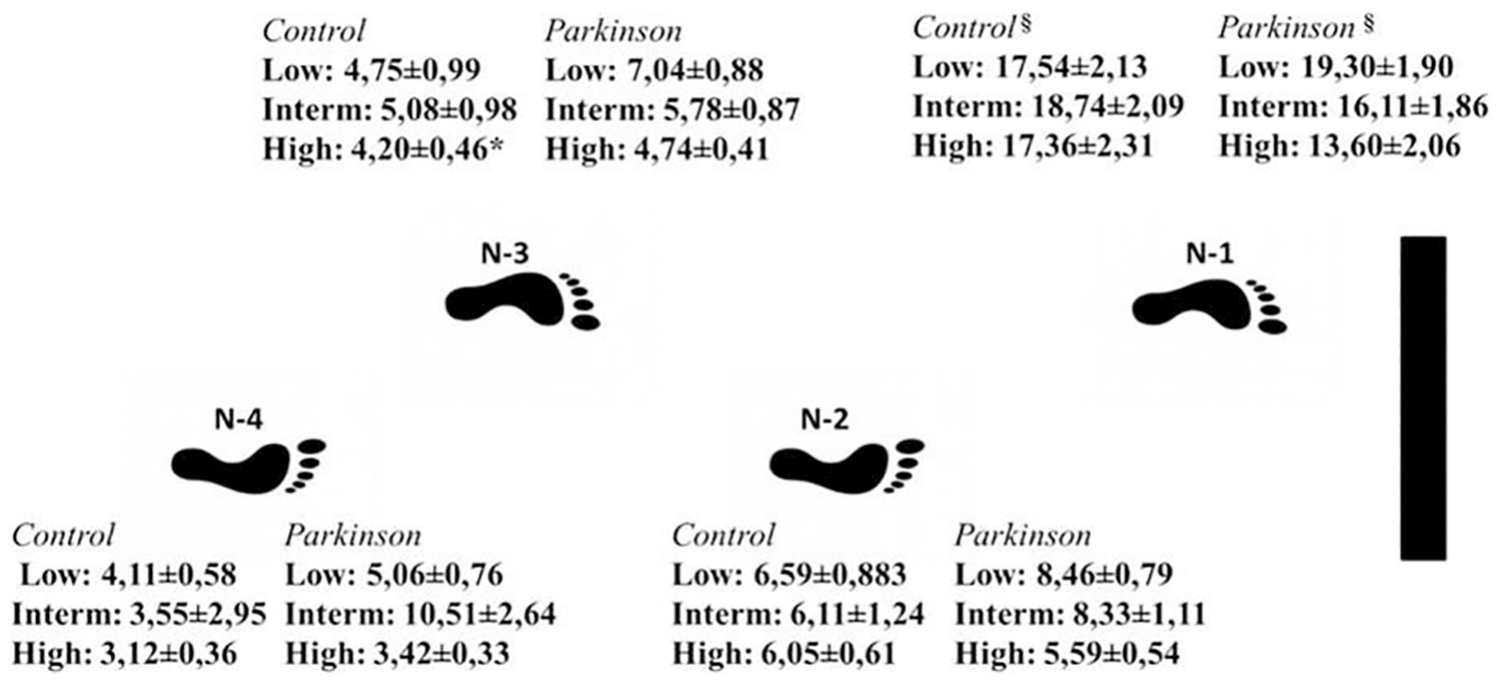
Means and standard deviations of variability of horizontal distance between the foot and obstacle in each step, for the conditions with the presence of an obstacle. §—difference between n-1 and all other analyzed steps.

The variability of the distance between the foot and obstacle for each step had no effect for group (Wilks' Lambda = 0.81, F_4,22_ = 1.25; p = 0.31) or interaction between factors (Wilks' Lambda = 0.54, F_8,18_ = 1.88; p = 0.12). There was a significant effect for condition (Wilks' Lambda = 0.43, F_8.18_ = 2.90; p<0.02). The participants increased variability of foot positioning in the n-1 step (Fig 3) in relation to other steps (n-2, n-3 and n-4 steps) in the presence of obstacles (low, intermediate and high obstacle conditions) during walking.

### Toe clearance

There was no significant effect for group (Wilk’s Lambda = 0.955, F_2,25_ = 0.588, p = 0.56). There was effect for obstacle height for leading limb toe-clearance (Wilk’s Lambda = 0.456, F_2,25_ = 13.381, p> 0.001) and trailing limb toe-clearance (Wilk’s Lambda = 0.456, F_2,25_ = 19.164, p> 0.001). For trailing limb, the toe-clearance during high obstacle was smaller than other heights (low—p = 0.007/ intermediate–p>0.001). For leading limb, the toe-clearance during high obstacle was greater than other heights (low—p = 0.05/ intermediate–p>0.001 –Table 4).

**Table 4:** Mean and standard deviation (in parenthesis) of toe-clearance for leading and trailing limb in each group.

	Control group		PD group	
	Trail limb	Leading limb	Trail limb	Leading limb
	TC	TC	TC	TC
**Low obstacle**	23.24	3.85	23.61	5.64
	(±5.07)	(±2.24)	(±7.94)	(±4.2)
**Intermediate obstacle**	19.51	3.63	20.23	3.5
	(±5.42)	(±1.42)	(±19.51)	(±2.66)
**High obstacle**	13.06*	7.84	14.72*	7.06
	(±5.9)	(±3.89)	(±8.77)	(±2.66)

TC—toe-clearance

*- difference between high obstacle and other obstacle heights.

## Discussion

The aim of this study was to investigate the effects of obstacle height on step-to-step and walking variability of spatial-temporal parameters in the approach phase of obstacle avoidance in older people with PD and neurologically healthy older individuals. For mean values of the spatial-temporal parameters, our findings corroborated with the literature [4,25,26]. The PD group demonstrated shorter step length and velocity, and longer double support duration. The disease characteristics may explain the deficits in gait parameters in older people with PD. Rigidity, hypometria and postural instability cause changes in walking pattern that affect gait performance in this population [4,26]. Furthermore, in people with PD, these adjustments are exacerbated when walking with obstacle avoidance [4] due to an increase in attentional demand and difficulty in coordinating sequential complex movements [27]. Thus, deficits caused by the disease and effects of the environment seem to impair gait in older people with PD.

Regarding variability of gait, our hypothesis was partially confirmed. For almost all spatial-temporal parameters during the approach phase to the obstacle, the PD group presented greater step-to-step and walking variability, confirming our hypothesis. However, surprisingly, the control group showed greater variability of step length and velocity during intermediate obstacle avoidance and greater variability of step width in the n-1 step when the participants were avoiding a low obstacle. Although unexpected, the greater variability in the control group during the approach phase for the intermediate obstacle avoidance seems to be an indication that obstacles of this height are common in the daily routine of the participants, such as stepping down from a curb in the street that has a height from 12 to 15 cm, according to the Brazilian Association of Technical Standards (ABNT). Therefore, the combination of their greater ability to avoid this obstacle height and a preserved neuromotor system [28] allowed neurologically healthy older people to feel comfortable about avoiding this obstacle so that performing adjustments during the approach phase increased the variability but without decreasing efficiency. In addition, individuals without neurological problems deal better with environment perturbation and can adapt [28]. Impaired systems (such as in older people with PD) do not have this adaptative capacity and require other elements to better adapt to the environment, such as visual and auditory cues, which increase the input to the cerebral circuitry impaired by the disease and improve gait [28]. This hypothesis is relevant, once studies with visual cues have demonstrate the importance and better results on gait analysis when this kind of cue is present [10,28]. On the other hand, intermediate obstacle avoidance disturbed the approach phase of the control group more than other obstacle heights. Low obstacle avoidance seemed to be an easier task for this population, which required fine (small) adjustments and did not increase the variability of the approach phase. High obstacle avoidance required more input from the neuromotor system and attention due to the increased danger of the task [29]. Therefore, the neurologically healthy older people preferred to maintain the adjustments during the trials, decreasing the risk of the task. In addition, high obstacles reduce the margin of adjustments during the task [15]. However, for intermediate obstacle avoidance, the individuals presented uncertainty as to which gait pattern to adopt and needed to adjust the pattern in each trial, increasing the variability. Furthermore, the participants maintain a safe elevation (~30cm—the sum of obstacle height with toe clearance). In this way, the participant chosed to maintain an pattern to avoid contantly alteration which could prejudicated obstacle avoidance becoming it more dangerous.

The main finding of our study was that low obstacle avoidance increased the variability of spatial-temporal parameters (both walking and, mainly, step-to-step variability) during the approach phase to the obstacle compared to other obstacle heights (intermediate and high obstacles). This finding contradicted our second hypothesis. In addition, there was an increase in spatial-temporal variability in the steps closer to the obstacle (n-2 and n-1 steps), especially the final step before the obstacle, in comparison to steps further from the obstacle (n-3 and n-4 steps), especially for the PD group, which confirmed our third hypothesis. Therefore, in the following paragraphs, two possible explanations for our findings are discussed separately. First, an explanation related to the greater variability in the PD group is presented (our first hypothesis). Second, interpretations are offered for the greater variability in low obstacle avoidance, especially for the variability in the final step before the obstacle avoidance.

### Greater variability of the approach phase in older people with PD

Obstacle avoidance increased the variability of spatial-parameters in the approach phase to obstacle avoidance in older people with PD. The behavior seemed to indicate that the PD group present impairments in both the mechanisms that regulate gait rhythm and the central pattern generator (related to higher walking and step-to-step variability of spatial parameters) and those that regulate balance (higher step-to-step variability of temporal parameters). Moreover, this exacerbated variability in older people with PD could be interpreted, from a pathophysiology aspect, as a deficit in the basal ganglia internal rhythmicity [30–35]. The basal ganglia are responsible for automatic behavior [10], which involves the circuitry of the motor cortex, striatum, globus pallidus, thalamus and supplementary motor area that are responsible for feedback and are related to internal control of repetitive movements, such as walking [10]. Any dysfunction in the basal ganglia circuitry, predominantly dopaminergic neurons in the posterior putamen and its nigrostriatal projection [10], is related to increase in gait variability, especially in spatial parameters. Thus, older people with PD need to reallocate the control of activities, which before the disease were “automatic”, to cortical areas, making the gait less automatic [10, 36,37], mainly in complex environments. Moreover, balance impairments in older people with PD seem to increase gait variability. Postural instability in this population requires adjustments of the center of mass trajectory from time-to-time, which seems to contribute to increased gait variability in older people with PD [4,10], considering that falls in PD are related to higher variability of stride length [12]. In addition, the difficulty that older people with PD have in performing two tasks at the same time, such as gait and obstacle avoidance, is an aspect to consider regarding greater variability of spatial-temporal parameters during the approach phase to an obstacle [9,10].

Substantial deficits in older people with PD such as gait deficits, visual problems, cognitive impairments, motor fluctuations [7,9,10] seem to exacerbate the impairments (greater variability, shorter step length, greater time on double support) during the approach phase to obstacle avoidance. Moreover, the motor system in individuals with PD presents impairments [27], requiring more time to plan the movement. In this way, the combination of these two aspects seems to impair the gait of this population, increasing the chances of falling. On the other hand, older people with PD may use non-impaired circuitry, such as the cortical-cerebellar circuitry [15]. Therefore, the groups could present similar behavior such as variability of the horizontal distance between the foot and the obstacle. This behavior reinforces that older people with PD use visual information (obstacle) to improve spatial control of steps [38] and decrease variability, similar to the control group.

#### Low obstacle avoidance increases variability of the approach phase, mainly for the final step (n-1)

Motor planning (and motor adaptations) was initiated much earlier in the approach phase for the higher obstacle conditions compared to the low obstacle condition, adjusting the approach phase in the final step before obstacle avoidance (n-1 step). Previous studies have indicated that obstacle height seems to influence the planning of obstacle avoidance [39]. Both groups planned the obstacle avoidance in advance (feedforward control) when they avoided higher obstacles compared to lower obstacles, which indicated greater variability in steps further from the obstacle (n-4 and n-3) for intermediate and high obstacle avoidance. This could be considered a safe strategy due to the individuals having time to readjust their walk during the final steps if necessary. However, planning of low obstacle avoidance seemed to take place in the final step before obstacle avoidance (online control), mainly for older people with PD. This strategy could be considered dangerous since the individuals do not have time to readjust their walk if the performed adjustment was inadequate. The majority of falls occur during the approach phase in the steps nearest the obstacle [29]. Deficits in environmental perception caused by aging [40] probably explain this finding, which seems to indicate that both neurologically healthy older individuals and older people with PD have a dangerous perception of the higher obstacles and do not perceive the same for the low obstacle, adjusting the approach phase to obstacle avoidance according to the height of the obstacle.

Despite important findings, this study has some limitations. The performance of the tasks in a block seems to favor the learning of the individual during the task. However, there was no trial effect on the experimental condition and the greater variability in the control group in some situations seems to contradict this limitation. Future studies may investigate the difference between PD subtypes (tremor dominant or postural instability gait deficit PD) Moreover, our PD patients were relatively mild without balance deficits. Perhaps this is a justification for testing while “Off” medication in future. Finally, the present study restricted approach phase dictating starting position and (right) lead limb crossing. Despite this manipulation was not informed to participants, the protocol used in the study should be taken into consideration when interpreting our results. Another important point that could have a little influence in our results it's how participants see and process the obstacle. For example, the high obstacle could induce participants process it before, once they could saw it earlier due to its height whereas the low obstacle they could perceive it later, leading to later adjustments. But, we just suppose this, once an eye tracker analysis is needed to affirm and we encourage further studies to perform this analysis.

## Conclusion

Obstacle avoidance during walking increased variability of spatial-temporal parameters of the approach phase in older people with PD and neurologically healthy older individuals. Older people with PD demonstrated greater variability of the approach phase than neurologically healthy individuals, with the exception of intermediate obstacle avoidance. In addition, the variability of spatial-temporal parameters of the approach phase was according to the height of the obstacle. Both groups changes motor planning (and motor adaptations) according to obstacle height and initiating changes much earlier in the approach phase for the higher obstacle conditions compared to the low obstacle condition. These findings could improve understanding about obstacle negotiation, mainly for low obstacles. Tripping is more common with low obstacles. Furthermore, understanding variability can provide insights into motor planning of individuals with PD during obstacle avoidance.

## Supporting information

S1 Data (XLSX)
